# A quantitative and qualitative study on the neuropsychiatric sequelae of acutely ill COVID-19 inpatients in isolation facilities

**DOI:** 10.1038/s41398-020-01039-2

**Published:** 2020-10-19

**Authors:** Fengyi Hao, Wilson Tam, Xiaoyu Hu, Wanqiu Tan, Li Jiang, Xiaojiang Jiang, Ling Zhang, Xinling Zhao, Yiran Zou, Yirong Hu, Xi Luo, Roger S. McIntyre, Travis Quek, Bach Xuan Tran, Zhisong Zhang, Hai Quang Pham, Cyrus S. H. Ho, Roger C.M. Ho

**Affiliations:** 1 The First People’s Hospital of Chongqing Liang Jiang New Area, Chongqing, China; 2 Alice Lee Centre for Nursing Studies, National University of Singapore, Singapore, Singapore; 3 First Affiliated Hospital of Chongqing Medical University, Chongqing, China; 4 National University of Singapore (Chongqing) Research Institute, Chongqing, China; 5 Department of Neurology, Daping Hospital, Army Medical University, Chongqing, 400042 China; 6 Mood Disorders Psychopharmacology Unit, University Health Network, University of Toronto, Toronto, Canada; 7 Department of Psychological Medicine, Yong Loo Lin School of Medicine, National University of Singapore, Singapore, 119077 Singapore; 8 Institute for Preventive Medicine and Public Health, Hanoi Medical University, Hanoi, 100000 Vietnam; 9 Bloomberg School of Public Health, Johns Hopkins University, Baltimore, MD 21205 USA; 10 Faculty of Education, Huaibei Normal University, Huaibei, 235000 China; 11 Institute for Global Health Innovations, Duy Tan University, Da Nang, Vietnam; 12 Faculty of Medicine, Duy Tan University, Da Nang, Vietnam; 13 Department of Psychological Medicine, National University Health System, Singapore, Singapore; 14 Institute for Health Innovation and Technology (iHealthtech), National University of Singapore, Singapore, 119077 Singapore

**Keywords:** Diseases, Depression, Psychiatric disorders

## Abstract

This study examined the neuropsychiatric sequelae of acutely ill patients with coronavirus disease 2019 (COVID-19) infection who received treatment in hospital isolation wards during the COVID-19 pandemic. Ten COVID-19 patients who received treatment in various hospitals in Chongqing, China; 10 age- and gender-matched psychiatric patients; and 10 healthy control participants residing in the same city were recruited. All participants completed a survey that collected information on demographic data, physical symptoms in the past 14 days and psychological parameters. Face-to-face interviews with COVID-19 patients were also performed using semi-structured questions. Among the COVID-19 patients, 40% had abnormal findings on the chest computed topography scan, 20% had dysosmia, 10% had dysgeusia, and 80% had repeated positivity on COVID-19 reverse-transcription polymerase chain reaction testing. COVID-19 and psychiatric patients were significantly more worried about their health than healthy controls (*p* = 0.019). A greater proportion of COVID-19 patients experienced impulsivity (*p* = 0.016) and insomnia (*p* = 0.039) than psychiatric patients and healthy controls. COVID-19 patients reported a higher psychological impact of the outbreak than psychiatric patients and healthy controls, with half of them having clinically significant symptoms of posttraumatic stress disorder. COVID-19 and psychiatric patients had higher levels of depression, anxiety and stress than healthy controls. Three themes emerged from the interviews with COVID-19 patients: (i) The emotions experienced by patients after COVID-19 infection (i.e., shock, fear, despair, hope, and boredom); (ii) the external factors that affected patients’ mood (i.e., discrimination, medical expenses, care by healthcare workers); and (iii) coping and self-help behavior (i.e., distraction, problem-solving and online support). The future direction in COVID-19 management involves the development of a holistic inpatient service to promote immune and psychological resilience.

## Introduction

The World Health Organization (WHO) declared the coronavirus disease 2019 (COVID-19) outbreak a pandemic on 11 March 2020^1^. As of May 30, the number of confirmed cases was more than six million, with the number of death cases at >366,000, and the number of recovered cases at more than two million worldwide^2^. The symptoms of COVID-19 include general symptoms, such as fever; chills and malaise; respiratory symptoms including cough, breathing difficulty and coryza; gastrointestinal symptoms including vomiting and diarrhea; and neurological symptoms including headache and giddiness^3^. In a recent report, some patients with COVID-19 complained of olfactory and taste disorders^4^.

Differential levels of psychological distress as a result of vicarious traumatization from COVID-19 were found in different groups of people, ranging from the general public^5^, to psychiatric patients^6^, individuals under quarantine^7^ and healthcare workers^8^. However, there is currently limited research on the neuropsychiatric sequalae and psychological impact of COVID-19 patients, with one study so far reporting that most clinically stable patients suffered from significant posttraumatic stress disorder (PTSD) symptoms^9^. The emotional and psychological needs of COVID-19 patients could be very much different from those with pre-existing psychiatric illnesses and people in the community.

Several hypotheses exist to explain why COVID-19 patients may suffer from neuropsychiatric ramifications. COVID-19 is postulated to infect the central nervous system^10^ via the peripheral trigeminal or olfactory nerves following intranasal inoculation^11^. It then invades regions of the cerebral cortex, basal ganglia, and midbrain that are closely linked to the olfactory bulb. From psychological perspectives, the perceived threat, susceptibility, and illness severity coupled with physical discomfort, loneliness and psychosocial stressors may evoke emotional disturbances, such as anger, fear, hysteria, depression, anxiety, and other psychological issues, in patients acutely infected with COVID-19. These psychological factors may in turn reduce innate immunity through cell-mediated immune activation via the release of several inflammatory markers, such as IL-6, IL-12, and tumor necrosis factor-alpha, which are implicated in the pathogenesis of depression^12^ and PTSD^13^. This therefore suggests the possibility of immune dysregulation as a shared pathogenesis in COVID-19 infection and psychiatric disorders. There has been an increasing number of qualitative studies that examined the in-depth effects of COVID-19 pandemic on the feelings, behavior, and attitude of healthcare workers^14^ and caregivers^15^. However, there is still a paucity of research on the psychological mechanisms and impact of COVID-19 on infected patients, and how they cope through the isolation period. This highlights a pertinent knowledge gap that needs to be addressed that is essential for holistic management.

The present study performed a quantitative evaluation of the neuropsychiatric sequelae of patients with acute COVID-19 infection who received treatment in the hospital isolation wards, and compared these patients with psychiatric patients and healthy controls during the COVID-19 pandemic. We hypothesized that COVID-19 patients would exhibit more neuropsychiatric symptoms than psychiatric patients and people in the community. We also performed face-to-face semi-structured interviews with patients to explore other possible symptoms that may be missed in quantitative studies^16^, understand their subjective experience, and the psychological impact of contracting COVID-19. Findings obtained from this study will be invaluable in setting up a service that can adequately address the biopsychosocial needs of the COVID-19 pandemic^6,17^.

## Methods

### Research design

The present study obtained the best quality data using face-to-face interviews with acutely ill COVID-19 patients during their hospitalization in various hospitals in Chongqing, China. The interviews were performed from 18 to 26 March 2020. Interviewers wore full personal protective equipment (PPE) when performing the interviews with COVID-19 patients in the isolation ward. Patients were interviewed while they were still COVID-19 positive in status and that they were on supportive treatment. A series of standardized validated questionnaires were used for the quantitative component of the study. For the qualitative component of the study, the interviewers performed semi-structured interviews using open-ended questions to examine patients’ perception and feelings during the current outbreak. Patients were encouraged to speak openly, highlight issues pertinent to them, and elucidate their responses with examples. Patients were allowed to withdraw their consent at any time during the interview. The interviews were audio-recorded and kept strictly confidential. Each interview took ~40–90 mins and the interviewers were instructed to remain neutral during the data collection process and establish a rapport with the patients using the techniques of acceptance, active listening and clarification to ensure the authenticity of the information and minimize bias. In the event that the patients became emotional during the interview, psychological support and intervention were provided to them.

Age- and gender-matched psychiatric patients and healthy control participants residing in the same city were recruited during the COVID-19 epidemic and used as a comparison. They did not undergo confirmatory COVID-19 testing as they did not have any symptoms that were suggestive of COVID-19 infection or had positive contact history, and the supplies of confirmatory kits in the hospital were limited. The Ethics Review Committee of The First People’s Hospital of Chongqing Liang Jiang New Area approved this project (IRB No. 2020-02-002). Informed consent was obtained from all subjects.

### Study participants inclusion and exclusion criteria

COVID-19 patients were aged 18 years or older and hospitalized during the time of assessment. The diagnosis of COVID-19 was made using reverse-transcription polymerase chain reaction (RT-PCR) testing and chest computed tomography (CT) for evaluation of COVID-19. To be included, patients could not have any pre-existing psychiatric illnesses or unstable medical conditions. Patients with severe complications requiring oxygen supplementation or who were medically unstable were excluded from the study.

The inclusion criteria were different for psychiatric patients and healthy controls. All the psychiatric patients were aged 18 years or older and were previously diagnosed by psychiatrists with F32, major depressive disorder—single episode; F33, major depressive disorder—recurrent episodes; F41, other anxiety disorders, including generalized anxiety disorder and panic disorder; and F41.8, mixed anxiety and depressive disorder, based on the 10th revision of the International Statistical Classification of Diseases and related Health Problems (ICD-10) criteria. Healthy control subjects were aged 18 years or older and did not have a history of psychiatric illnesses. The exclusion criteria included the presence of chronic medical disorders, including neurological, cardiovascular, respiratory, endocrine and inflammatory disorders, or suspected or confirmed cases of COVID-19.

### Outcomes

The structured questionnaire covered several areas: (i) demographic data; (ii) physical symptoms and self-rated physical health status in the past 14 days; (iii) Impact of Event Scale-Revised (IES-R), (iv) Depression, Anxiety and Stress Scale (DASS-21), (v) the Insomnia Severity Index (ISI), and (vi) other psychiatric symptoms.

Sociodemographic data were collected regarding gender, age, education, and household size. Physical symptom variables in the past 14 days included fever, chills, headache, myalgia, cough, difficulty breathing, dizziness, coryza, sore throat, persistent fever, nausea, vomiting and diarrhea. Respondents were asked to rate their physical health status.

The psychological impact of the COVID-19 outbreak was measured using the IES-R. The IES-R is a self-administered questionnaire that is well-validated in the Chinese population for determining the extent of the psychological impact after exposure to a public health crisis within one week of exposure^18^. This 22-item questionnaire is composed of three subscales and aims to measure the mean avoidance, intrusion and hyperarousal^19^. The total IES-R score was divided into 0–23 (normal), 24–32 (mild psychological impact), 33–36 (moderate psychological impact) and >37 (severe psychological impact). Mental health status was measured using the Depression, Anxiety and Stress Scale (DASS-21), and the scores were calculated based on previous studies^20^. The total depression subscale score was divided into normal (0–9), mild depression (10–12), moderate depression (13–20), severe depression (21–27) and extremely severe depression (28–42). The total anxiety subscale score was divided into normal (0–6), mild anxiety (7–9), moderate anxiety (10–14), severe anxiety (15–19), and extremely severe anxiety (20–42). The total stress subscale score was divided into normal (0–10), mild stress (11–18), moderate stress (19–26), severe stress (27–34) and extremely severe stress (35–42). The DASS is a reliable and valid measure for assessing the mental health of the Chinese population^21,22^. Both the IES-R and DASS were previously used in research related to the COVID-19 pandemic^5–8^. The sleep quality of respondents was measured using the Insomnia Severity Index (ISI)^23^. The ISI has seven questions that are summed to produce a total score. The total ISI score was divided into no clinically significant insomnia (0–7), subthreshold insomnia (8–14), moderately severe clinical insomnia (15–21) and severe clinical insomnia (22–28).

As for the semi-structured interviews for COVID-19 patients, eight questions were included: (i) describe a situation in which you experienced negative emotions (unpleasant feelings) during the outbreak and how you felt and thought at the time; (ii) describe a situation in which you experienced positive emotions during the outbreak and how you felt and thought at the time; (iii) how did you feel and react after your first infection (thinking, feelings, behavior, any bodily sensation)?; (iv) how did you feel and react after you were tested positive again (thinking, feelings, behavior, any bodily sensation)?; (v) what worries do you have about the future?; (vi) what enlightenment will this experience bring to your life and why is that so?; (vii) what advice, if any, would you give to countries and people experiencing a new epidemic?; and (viii) what do you think you, your family and friends did to help you recover physically and emotionally? All the interviews were audio-recorded with prior consent given by the patients. The audio recordings were transcribed within 24 hours of each interview and reviewed by the interviewers to safeguard accuracy. As the interviews, transcriptions, and analyzed data were in Chinese, they were translated into English by some study team members and back translated by another group of study team members to ascertain that the meaning was retained.

### Data analysis

Descriptive statistics were used to summarize the variables, means and standard deviations were used for continuous variables, and frequencies and percentages were used for categorical variables. Inferential statistics, including the independent sample *t* test and Pearson’s chi-squared test, were used to examine differences in the outcome variables between the psychiatric patient and healthy subject groups. Multiple linear regression with a backward selection method was used to examine the association between the outcome variables, the two groups of subjects and the demographic variables. All analyses were performed using IBM SPSS Statistics 22, and the level of significance was set at 5%. As for the qualitative component of the study, the interview was transcribed, and a detailed reading and re-reading of the transcripts was performed to identify significant key words and phrases. Understanding and validating the expressed meanings of these words and phrases were done through research team meetings to arrive at a consensus. Then, codes were assigned and arranged into themes and sub-themes using the concepts of grounded theory for data analyses.

## Results

### Sociodemographic and physical variables

The sociodemographic characteristics of the patients and healthy control participants are presented in Table 1. COVID-19 and psychiatric patients were age- and gender-matched with healthy subjects. Patients significantly differed from healthy controls with regards to the physical symptoms experienced (*p* = 0.014), with a higher proportion of COVID-19 patients than psychiatric patients and healthy controls reporting at least one symptom (60% vs 30% vs 0%). There was no difference between the three groups in self-reported health status.

**Table 1:** Demographic variables and health status of participants (*n* = 30).

Demographic variable	COVID-19 patients (*n* = 10)	Psychiatric patients (*n* = 10)	Healthy subjects (*n* = 10)	*P*
*Gender*				*1*
Male	6 (60%)	6 (60%)	6 (60%)	
Female	4 (40%)	4 (40%)	4 (40%)	
Age	37.4 (12.6)	37.4 (12.4)	37 (11.9)	0.996
*Education level*				*0.571*
Primary	0 (0%)	0 (0%)	0 (0%)	
Lower Secondary	2 (20%)	1 (10%)	4 (40%)	
Upper Secondary	3 (30%)	4 (40%)	4 (40%)	
Territory	3 (30%)	5 (50%)	1 (10%)	
Undergraduate	0 (0%)	0 (0%)	1 (10%)	
Postgraduate	2 (20%)	0 (0%)	0 (0%)	
*Household size*				*0.533*
1	0 (0%)	0 (0%)	2 (20%)	
2	1 (10%)	1 (10%)	1 (10%)	
3–5	8 (80%)	8 (80%)	7 (70%)	
6 or above	1 (10%)	1 (10%)	0 (0%)	
*Physical symptoms*				*0.014*
No symptoms	4 (40%)	7 (70%)	10 (10%)	
At least one symptom	6 (60%)	3 (30%)	0 (0%)	
*Self-reported health status*				*0.263*
Poor or worse	1 (10%)	1 (10%)	0 (0%)	
Normal	2 (20%)	6 (60%)	3 (30%)	
Healthy or better	7 (70%)	3 (30%)	7 (70%)	

All of the COVID-19 patients were clinically stable without the need for respiratory support. They were given supportive treatment and there was no clinical indication for any specific intervention. 40% of patients had abnormalities on chest CT, 20% had dysosmia, and 10% had dysgeusia. 80% of patients had repeat positivity for COVID-19 in RT-PCR testing and had been in isolation for 2 months when interviewed. The remaining 20% of patients were in isolation few days prior to the interview.

### Psychological symptoms

The psychological symptoms experienced by the study participants in the last 7 days are presented in Table 2. COVID-19 and psychiatric patients were significantly more worried about their health than healthy controls (*p* = 0.019), with more COVID-19 patients reporting moderate levels of worry (40%) and some psychiatric patients reporting serious level of worry (20%). Although the three groups were not statistically significant in regard to the parameter of discrimination, more COVID-19 patients reported feeling discriminated, ranging from a mild to very serious level (*p* = 0.160). More COVID-19 patients were impulsive as compared with psychiatric patients and healthy controls (*p* = 0.016), with half of the COVID-19 patients experiencing a mild level of impulsivity, and 20% of psychiatric patients experienced moderate to very serious levels of impulsivity.

**Table 2:** Psychological symptoms experienced by participants in last 7 days (*n* = 30).

Psychological symptoms	COVID-19 patients (*n* = 10)	Psychiatric patients (*n* = 10)	Healthy subjects (*n* = 10)	*P*
*Felt fine, no problem*				*0.501*
No	4 (40%)	8 (80%)	6 (60%)	
Mild	2 (20%)	1 (10%)	3 (30%)	
Moderate	2 (20%)	0 (0%)	1 (10%)	
Serious	1 (10%)	1 (10%)	0 (0%)	
Very serious	1 (10%)	0 (0%)	0 (0%)	
*Felt stressful*				*0.281*
No	4 (40%)	3 (30%)	4 (40%)	
Mild	1 (10%)	5 (50%)	4 (40%)	
Moderate	3 (30%)	1 (10%)	2 (20%)	
Serious	2 (20%)	1 (10%)	0 (0%)	
Very serious	0 (0%)	0 (0%)	0 (0%)	
*Felt irritability*				*0.168*
No	4 (40%)	3 (30%)	4 (40%)	
Mild	1 (10%)	6 (60%)	4 (40%)	
Moderate	4 (40%)	0 (0%)	2 (20%)	
Serious	1 (10%)	0 (0%)	0 (0%)	
Very serious	0 (0%)	1 (10%)	0 (0%)	
*Felt anxious*				*0.207*
No	3 (30%)	5 (50%)	5 (50%)	
Mild	2 (20%)	4 (40%)	4 (40%)	
Moderate	3 (30%)	0 (0%)	1 (10%)	
Serious	2 (20%)	0 (0%)	0 (0%)	
Very serious	0 (0%)	1 (10%)	0 (0%)	
*Felt upset*				*0.309*
No	5 (50%)	6 (60%)	5 (50%)	
Mild	1 (10%)	2 (20%)	4 (40%)	
Moderate	4 (40%)	1 (10%)	1 (10%)	
Serious	0 (0%)	0 (0%)	0 (0%)	
Very serious	0 (0%)	0 (0%)	0 (0%)	
*Felt meaningless*				*0.201*
No	7 (70%)	9 (90%)	10 (100%)	
Mild	1 (10%)	0 (0%)	0 (0%)	
Moderate	2 (20%)	0 (0%)	0 (00%)	
Serious	0 (0%)	1 (10%)	0 (0%)	
Very serious	0 (0%)	0 (0%)	0 (0%)	
*Felt without interest to do everything*				*0.796*
No	6 (60%)	7 (70%)	7 (70%)	
Mild	3 (30%)	1 (10%)	2 (20%)	
Moderate	1 (10%)	1 (10%)	1 (10%)	
Serious	0 (0%)	1 (10%)	0 (0%)	
Very serious	0 (0%)	0 (0%)	0 (0%)	
*Worried about health*				*0.019*
No	3 (30%)	5 (50%)	3 (30%)	
Mild	3 (30%)	3 (30%)	7 (70%)	
Moderate	4 (40%)	0 (0%)	0 (0%)	
Serious	0 (0%)	2 (20%)	0 (0%)	
Very serious	0 (0%)	0 (0%)	0 (0%)	
*Felt being discriminated*				*0.16*
No	5 (50%)	10 (100%)	9 (90%)	
Mild	3 (30%)	0 (0%)	1 (10%)	
Moderate	1 (10%)	0 (0%)	0 (0%)	
Serious	0 (0%)	0 (0%)	0 (0%)	
Very serious	1 (10%)	0 (0%)	0 (0%)	
*Heard voices*				*0.355*
No	9 (90%)	10 (10%)	10 (100%)	
Mild	1 (10%)	0 (0%)	0 (0%)	
Moderate	0 (0%)	0 (0%)	0 (0%)	
Serious	0 (0%)	0 (0%)	0 (0%)	
Very serious	0 (0%)	0 (0%)	0 (0%)	
*Felt being followed*				*0.117*
No	10 (100%)	8 (80%)	10 (100%)	
Mild	0 (0%)	2 (20%)	0 (0%)	
Moderate	0 (0%)	0 (0%)	0 (0%)	
Serious	0 (0%)	0 (0%)	0 (0%)	
Very serious	0 (0%)	0 (0%)	0 (0%)	
*Felt impulsive*				*0.016*
No	5 (50%)	8 (80%)	10 (100%)	
Mild	5 (50%)	0 (0%)	0 (0%)	
Moderate	0 (0%)	1 (10%)	0 (0%)	
Serious	0 (0%)	0 (0%)	0 (0%)	
Very serious	0 (0%)	1 (10%)	0 (0%)	
*Drank too much*				*0.355*
No	9 (90%)	10 (100%)	10 (100%)	
Mild	1 (10%)	0 (0%)	0 (0%)	
Moderate	0 (0%)	0 (0%)	0 (0%)	
Serious	0 (0%)	0 (0%)	0 (0%)	
Very serious	0 (0%)	0 (0%)	0 (0%)	
*Had suicidal ideations*				*0.396*
No	9 (90%)	9 (90%)	10 (100%)	
Mild	1 (10%)	1 (10%)	0 (0%)	
Moderate	0 (0%)	0 (0%)	0 (0%)	
Serious	0 (0%)	0 (0%)	0 (0%)	
Very serious	0 (0%)	0 (0%)	0 (0%)	
*Had thoughts of hurting others*				*0.396*
No	9 (90%)	9 (90%)	10 (100%)	
Mild	1 (10%)	0 (0%)	0 (0%)	
Moderate	0 (0%)	1 (10%)	0 (0%)	
Serious	0 (0%)	0 (0%)	0 (0%)	
Very serious	0 (0%)	0 (0%)	0 (0%)	

### Psychological impact

The psychological impact of the COVID-19 pandemic on participants are presented in Table 3. The psychological impact as measured using the IES-R scale, revealed that the COVID-19 patients had a higher mean score (22.3, SD = 16.3) than psychiatric patients (18.4, SD = 19.4) and healthy controls (14.7, SD = 7.9), although there was no significant difference between the three groups. A greater proportion of COVID-19 patients (50%) than the other two groups experienced a psychological impact of at least mild and above severity in keeping with clinically significant symptoms of PTSD, as indicated by IES score of 24 and above, although this result was not statistically significant. COVID-19 and psychiatric patients had higher DASS-21 sub-scores of anxiety, depression and stress than healthy controls, with higher scores of anxiety (6.4 vs 5.0) and depression (7.8 vs 7.0) for psychiatric patients than for COVID-19 patients. The DASS-21 sub-score of stress was higher for COVID-19 patients than for psychiatric patients (9.2 vs 7.8). All three *p* values were however insignificant. There were also no significant differences in the severity dichotomization of anxiety, depression, and stress among the three groups. For sleep quality, a higher proportion of COVID-19 patients (50%) reported insomnia than psychiatric patients (30%) and healthy controls (0%), and this difference was statistically significant (*p* = 0.039).

**Table 3:** Psychological impact of participants (*n* = 30).

	COVID-19 patients (*n* = 10)	Psychiatric patients (*n* = 10)	Healthy subjects (*n* = 10)	*P*
IES Total	22.3 (16.3)	18.4 (19.6)	14.7 (7.9)	0.551
*IES*				*0.24*
0–23	5 (50%)	8 (80%)	8 (80%)	
24 or more	5 (50%)	2 (20%)	2 (20%)	
DASS-21 (anxiety)	5.0 (4.3)	6.4 (10.2)	0.8 (1.4)	0.149
*DASS-21 (anxiety)*				*0.161*
No (0–7)	7 (70%)	8 (80%)	10 (10%)	
Mild (8–9)	2 (20%)	0 (0%)	0 (0%)	
Moderate or above (10+)	1 (10%)	2 (20%)	0 (0%)	
DASS-21 (depression)	7.0 (6.0)	7.8 (10.3)	1.0 (1.4)	0.075
*DASS-21 (depression)*				*0.24*
No (0–9)	6 (60%)	8 (80%)	10 (100%)	
Mild (10–13)	1 (10%)	1 (10%)	0 (0%)	
Moderate or above (14+)	3 (30%)	1 (10%)	0 (0%)	
DASS-21 (stress)	9.2 (5.8)	7.8 (10.6)	2.2 (2.7)	0.087
*DASS-21 (stress)*				*0.396*
No (0–14)	9 (90%)	9 (90%)	10 (100%)	
Mild (15–18)	1 (10%)	0 (0%)	0 (0%)	
Moderate or above (19+)	0 (0%)	1 (10%)	0 (0%)	
ISI	8.0 (6.6)	8.1 (8.7)	3.4 (2.5)	0.196
*ISI*				*0.039*
0–7	5 (50%)	7 (70%)	10 (100%)	
8 or above	5 (50%)	3 (30%)	0 (0%)	

### Qualitative psychological data

Three main themes emerged from the COVID-19 patients’ accounts of their experiences of the outbreak: (i) emotions of the patients after COVID-19 infection; (ii) external factors that affected patients’ mood; (iii) coping and self-help behavior. Each main theme and its sub-themes are discussed and illustrated in some of the participants’ verbatim accounts.

#### Theme 1: emotions of the patients after COVID-19 infection

##### 1.1. Emotions generated by the infection

All patients reported that the COVID-19 infection was a major stress event for them. This stress was particularly due to the high infectivity and potential lethality of the viral infection. Emotional disturbances occurred in patients, especially in the initial period after diagnosis with the infection, and some of the first emotions included surprise, fear, bewilderment, and questioning why the virus had infected them.

*“I feel so scared and stressed. I’m afraid that I’m going to break down and I won’t be able to hold on anymore.” (Patient 9)*

*“I was actually quite surprised I got infected because I did a good job protecting myself… I had no contact with anyone from foreign countries, I only went downstairs to buy daily necessities twice in 14 days, and I wore a mask the whole time. So, I was surprised to find out I was infected.” (Patient 10)*

After the initial realization and acceptance of contracting the disease, patients began to feel depressed, and some individuals had desperate thoughts, such as the fear of being unable to leave the hospital or of dying.

*“When the doctor told me that I was positive with the virus, at that moment, I thought it must be very serious. Would I be able to leave the hospital alive?” (Patient 7)*

However, once past the initial phases, some patients accepted their predicament and adjusted their mindset to receive treatment.

*“At first, I was very worried and felt miserable…then I stopped thinking about these because I knew it was meaningless to think about them now. The key was to get well quickly.” (Patient 6)*

Patients who tested positive for COVID-19 repeatedly during their stay in the hospital might have experienced a sense of hopelessness and helplessness that their chances for recovery had been “dashed”. This feeling might have triggered previous fears and worries of their condition deteriorating. There was also increased stress of waiting for repeated test results. With the absence of a viable cure and the limited understanding of the consequences of the novel viral infection, there was much apprehension, and some patients diverted excessive attention to their body, which led to the manifestation of somatic symptoms.

*“I have always been a little worried about my health. But now, my concerns about my health are more serious. I previously had high levels of uric acid, so I stopped eating meat or drink any soup… despite the doctor telling me that it is actually appropriate to eat. In the hospital, I don’t eat the apples that they provide me at night… I think they are cold, and it is bad to eat at night. I also don’t let my wife eat fruits at night. I’m worried that my illness is because of my underlying poor health constitution.” (Patient 1)*

With the stabilization of their disease, patients started worrying about people close to them and also longer-term issues, such as employment, financial problems and vaccine development.

*“I’m most worried about my children. I have been in isolation for so long. One of my children is still very young, and he is used to sleeping with me every day. I wonder if he is able sleep well now?” (Patient 5)*

*“…Wait for the country to develop a vaccine. Do we have to wait half a year to know the results of the human experiment? I have antibodies from the infection. I’m not afraid. I don’t need a vaccine.” (Patient 2)*

Some individuals had a relatively stable mental state, which may be related to the psychological resources of their life experiences (such as military career) and their preconceived ideas about diseases and viruses (such as being healthy and not afraid of any diseases).

*“I have a positive mentality… haha. I used to be in the military, so being stuck in one place for two months is nothing to me. I can manage myself well, be it my daily living or mentality.” (Patient 4)*

*“As long as there is no defect in my body, the disease should not be taken seriously. How many epidemics were there before this one? For instance, the 1918 Spanish flu pandemic that spread across the world. A century has passed. How is this pandemic different from the Spanish flu? In 1978, we went to fight the Da Hinggan Mountain fire. There’s also the great flood in 1998 in which the Jiujiang dike was washed down, and the SARS in 2003… There are a lot of disasters out there.” (Patient 8)*

##### 1.2. Emotions generated during hospitalization and isolation

The long hours that patients spent alone in a closed environment with their freedom limited may have led to a feeling of isolation, boredom, and a depressed mood.

*“It is too boring staying here, and it is making me irritable and restless, which I wasn’t before. If I test positive again, I’m going to go crazy. And I’ll find a way to get out somehow….” (Patient 7)*

*“The accommodation conditions in the last hospital was very limited. After my neighboring patients were discharged, I was alone in the room, with weak cell phone signal and no TV, so I was really bored. Every day I wonder - when will this life ever end?” (Patient 1)*

#### Theme 2. External factors affecting patients’ mood

During this unprecedented time of the COVID-19 pandemic, and living in a relatively special environment, various events might have affected the patients’ mood.

##### 2.1 Positive events affecting mood

The provision of systematic and sound national policies on protective and supportive measures for citizens might have mitigated the psychological burden of patients. Patients felt their own safety was guaranteed when the country that they lived in demonstrated it cares about the well-being of its people.

*“The government has exempted the medical expenses of patients suffering from this disease, so that people can concentrate on recovering without worries. The government has developed the right policies to guide us in the fight against the epidemic, and we are moving towards the right path. This shows the merits of the whole country in being able to focus on big things. What is there to worry about living in such a country and with such people?” (Patient 4)*

Furthermore, the care and warmth of the medical staff experienced by the patients made them realize that their feeling of isolation was due solely to the virus and not due to a lack of love and comradeship. The care and assistance provided by the medical staff made patients feel solidarity and warmth between people.

*“The nurse sister made me feel a lot of warmth every day… telling me to take my medicine and checking my temperature. During my illness, I learned that everyone was helping and supporting each other in need.” (Patient 4)*

*“The nurses are like angels. One day, I was hungry before the scheduled mealtime as I had diarrhea prior. I asked for a bowl of noodles, and the nurse immediately brought me noodles. The medical staff taught us to dance and sing… it was really fun, and we learned a lot. These nurses are very young in their 20* *s to 30* *s, and they haven’t been home for more than 30 days… it’s pretty hard for them too.” (Patient 6)*

Despite the inability to physically meet, family members and friends also provided psychological support and motivation to the patients via remote communication. Patients were also able to more deeply appreciate the value of kinship and friendship in the midst of this outbreak.

*“I am very grateful to my wife for her continued encouragement. During the period when I was staying in isolation, I felt lonely and sad. Due to changes in the environment, my state was worse than before, but she was always there encouraging me.” (Patient 2)*

*“I received a bunch of flowers and a letter from my friend. The content of the letter was simple and reassuring, which moved and made me feel good. I did not expect to encounter so many heartening moments during my stay in the hospital.” (Patient 5)*

##### 2.2 Negative events affecting mood

Research on COVID-19 is still nascent with many knowledge gaps. There is much panic about the infectivity and lethality of the virus. Patients reported discrimination and abuse by non-infected individuals. Excessive worry for family members also increased the psychological burden of patients.

*“After being discharged from the hospital the last time, we returned to our neighborhood and continued to be quarantined at home. Somehow our private information including our address and telephone number was leaked. Residents in the neighborhood said a lot of nasty things in the neighborhood WeChat group and regarded us as monsters. People who live in the same building are very worried about getting infected merely by passing by our door. In the group chat, they even talked about their fear to go downstairs. In the future, when we want to visit your clinic for review, I wonder if you will also avoid us?” (Patient 1)*

*“…He (family member) calls me every day and then passed the negativity to me. He feels better after talking to me, but it is actually hard for me. I absorbed his negative emotion and passed positive emotions to him.” (Patient 3)*

In addition to the negative comments of other people, patients spent more time on the Internet due to the lack of face-to-face communication opportunities during their stay in the hospital. When they saw negative news on the Internet, such as foreign communities’ lack of understanding of the Chinese measures and discrimination against Chinese people, they also felt angry and upset.

*“I have some foreign friends who believe that China’s reaction to the outbreak is an overkill and overly paranoid. Perhaps they underestimated the epidemic or they have never experienced the situation in Wuhan. They think that China is making a mountain out of a molehill and its measures are ridiculous. There are those who laugh at our policies and precautionary measures. These make me feel uncomfortable.” (Patient 9)*

*“From the beginning of the outbreak, leaders of some countries claimed that the virus originated from China and that China spread the virus across the world. As a result, the Chinese are loathed by other ethnic groups overseas. Many of China’s efforts to combat the outbreak have been deliberately ignored. I felt hated, discriminated against and that my life is threatened.” (Patient 10)*

Due to the isolation, patients felt anxious and worried about their unfinished matters. Their original social role was replaced by the patient sick role, which caused them to feel bored, irritated, and depressed.

*“I have been living here for two months, and I feel that there is no longer meaning to life. Sometimes I feel that my life and memories are not real, and I don’t know what I can achieve in the future. I used to be a person who’s very active with activities.” (Patient 6)*

*“Over the past two months, I have to pay my house mortgage, and my small business has been affected by the economic situation. My children have to go to school… My son is still very young… I worry that when I am not around, he cannot take care of himself.” (Patient 7)*

#### Theme 3. Coping and self-help behavior

In the face of stressful events, such as the pandemic, patients sought ways to adjust their emotions to help themselves. First, they comforted themselves by believing that the government and the medical staff would do their best to help them.

*“I try not to think too much. I constantly tell myself that young people such as myself don’t have medical illnesses, so COVID-19 will have a very small impact on me. I have confidence in my body, so I don’t have to worry so much, and this is the most important.” (Patient 3)*

*“I will always encourage myself to think positively and try my best to adjust my mindset. An integral part of recovery is the mental attitude, so psychologists play a key role in the process. (Patient 5)*

In addition to comforting themselves, patients also tried to maintain a regular routine that included proper exercises and an adequate mastering of disease knowledge to feel more empowered and in control.

*“I am one of the younger patients in isolation. At present, the inpatient isolated lifestyle requires one to go to bed and wake up early, and doing some gentle physical exercises planned by the ward team, which are more for middle-aged people and not particularly suitable for me… I have to find ways to enrich my life. Other people whom I know who are also in isolation are actively looking for ways to adjust.” (Patient 9)*

Patients also attempted to divert their attention from their situation via other activities.

*“Distraction is a great way, whether it’s talking to someone or finding something to do on your own. For example, when I was first admitted to the hospital, I always felt that I had breathing difficulties. But when I talked to you on the phone, I didn’t feel any difficulty at all. When I get distracted, the physical symptoms disappear…the more attention I pay to my breathing, the more obvious the shortness of breath becomes.” (Patient 5)*

With limited interpersonal communication during isolation, some patients created ways to make friends with other patients to expand their social contact and comfort one another.

*“When I just got admitted, I remembered walking along a corridor and seeing the door of a ward open. There was a fellow patient inside. I said: “Friend, let’s add each other on WeChat.” At that time, I needed friends, someone to understand me, to talk to me.” (Patient 7)*

## Discussion

Our study sample of COVID-19 patients had mild–moderate severity with less than half of them having abnormal findings on the chest CT. The paucity of abnormal findings on chest CT could possibly be due to an earlier pick-up rate with a shorter time after the onset of symptoms^24^. Furthermore, only a small proportion of them had olfactory and gustatory dysfunction. There has been increasing reports of smell and taste alterations as concurrent symptoms of COVID-19 infection. Interestingly, those with dysnomia and dysgeusia were found to have less–severe manifestations of other symptoms and tended to recover more quickly^11^, raising the possibility of olfactory and gustatory dysfunctions as potential markers of structural or functional morbidities^25^. The majority of our patients were retested positive for COVID-19. Although the exact causes of this phenomenon remain uncertain, there is a possibility that the viral infection can provoke an inflammatory milieu that results in aberrant immune responses. Such immune responses may trigger the propagation of host antibodies and lymphocytes that cross-react with both viral antigen and self-antigen, causing persistent infections, and potentiate the development of autoimmune neuropsychiatric sequelae particularly impulsivity and insomnia^26,27^.

A higher proportion of COVID-19 patients was found in our study to be impulsive as compared to psychiatric patients and healthy controls. These feelings might have been related to patients staying in isolation rooms for a prolonged duration with limited social interaction, lack of stimulation and loss of freedom, which may result in anger, fear, restlessness, and irritability. Staying in isolation rooms can negatively impact psychological well-being, in which previous studies highlighted higher scores for anger-hostility^28^, in addition to depression, anxiety, fear, and loneliness^29,30^. The acute stress experienced by patients can activate immune system responses via amplification of the corticotropin-releasing factor system that regulates impulsivity^31^ and releases pro-inflammatory cytokines such as IL-6 and TNF- α that evoke behavioral changes aimed to protect self from injury or harm^32^.

Sleep disturbances was also a prominent feature found in COVID-19 patients compared with psychiatric patients and healthy controls. This finding was consistent with other studies that found sleep problems occurring in people with naturally occurring respiratory infections^33^. Physical stress inflicted on the body by the infection coupled with psychological stress trigger off a cascade of system responses including cortisol release from the hypothalamic–pituitary–adrenal axis, and catecholamine, norepinephrine, and epinephrine release by the peripheral sympathetic-adrenomedullary system^34^. These systems in turn stimulate pro-inflammatory cytokine release targeting sleep-related functions among others such as metabolic and cardiovascular changes. Although these physiological mechanisms in the acute phase facilitate stress adaptation to “fight-or-flight” in the event of adversity, chronic activation of these mechanisms may cause detrimental bodily and psychological effects, such as obesity, depression, and even elevating the risk of developing a rhinovirus infection^35^.

Higher levels of depression, anxiety, stress, and PTSD were found in COVID-19 patients than healthy people, though in our study, these results were not statistically significant. Several reasons for the depressive and anxiety symptoms in our study include the perception of vulnerability to the virus, uncertainty and fear about the consequences of the infection, treatment outcome and death in the absence of a definitive treatment, the inability to resume their routine activities, worries about unfinished matters and loved ones, worries about their financial situation and stigma. Cognitive features of depression such as helplessness and hopelessness^36,37^, and somatic symptoms^38^ as part of the anxiety spectrum with hypervigilance on bodily sensations were also exemplified in the interviews. As for PTSD symptoms in COVID-19 patients, many expressed concerns about their health, family, livelihood, and future, which was disrupted, and displayed emotions that resembled the various stages of grief, ranging from denial, anger, bargaining, and depression to acceptance. With the possible shared immune dysregulation pathogenesis of COVID-19 and psychiatric disorders with bidirectional implications, it may be worthwhile to stabilize each condition to prevent worsening of the other, and medications that modulate the immune system to counteract COVID-19 could potentially be used as an antidepressant and vice-versa^39^. Anti-inflammatory drugs have been found to have antidepressant effects in clinical trials^12^, whereas antidepressants are also found to reduce central and peripheral levels of IL-1β with the alleviation of depressive symptoms^40^, suggesting their anti-inflammatory properties. Antidepressants with its relatively safe side-effect profile and affordability than other immunomodulators could therefore be a potential new therapeutic candidate in the treatment of COVID-19^41^. Psychiatric patients on the other hand, had higher scores of depression and anxiety than COVID-19 patients, which may be owing to their pre-existing poorer adaptive coping to acute stressful events. This result also raised the concern that more psychological support should be rendered to psychiatric patients despite their lack of infection; and psychiatric patients if infected with COVID-19, could be at higher risk of complications considering their emotional state and lowered immunity status. It is important to reiterate that we were unable to measure the inflammatory markers of participants and correlate them with the psychological parameters in our study, and owing to the cross-sectional nature of the study, it would not be possible to elucidate the causal relationship between mental illness and the inflammatory effects of COVID-19 infection. Nevertheless, this is an important research area that warrants further investigation.

There is a wide spectrum of emotions experienced by patients, and the heterogeneity in disease response could be contributed by various psycho-socio-economic factors, pre-existing medical comorbidities, differences in the immune system maturity that underline the immune response differences to the infection, temperament, and attachment styles^42,43^. Those with pre-existing chronic medical conditions and older adults may have lower innate immunity with greater susceptibility to the virus and risk having worse outcomes. Anxious, depressive, and cyclothymic temperaments as well as the insecure-anxious attachment dimension temperament have been found to predict psychological distress, whereas secure and avoidant attachment styles are protective^43^. Shock, fear, despair, and hope as found in our study are well-recognized symptoms of traumatization^44^, whereas boredom in relation to restlessness and irritability are especially relevant for those in isolation and quarantine. Boredom if entrenched and severe, can manifest as a neurotic disorder that may erode self-control, leading to impulsivity, and risk-taking behavior^45^.

Feeling discriminated was a pertinent issue encountered by COVID-19 patients, which is consistent with previous studies that highlighted the stigma experienced by infected patients^46^ who may be shunned by their loved ones, friends, and communities for being a carrier of the virus, or as part of a nationality or ethnic group that contributed to its transmission^47^. In addition, internalized stigma may also occur in which infected people view themselves as inferior to others, which leads to self-loathing as a result of their disease status^48,49^. Discrimination and stigmatization often lead to feelings of abandonment and loneliness^50^, which are further compounded in COVID-19 patients by staying in an isolation room and may last beyond discharge from the hospital^51^.

In our study, COVID-19 patients utilized the tripartite framework of positive coping style, cognitive appraisal, and social support to mitigate stress, promote positive emotions, and enhance perceived self-efficacy^52^. These factors facilitate psychological adaptation and resilience in the backdrop of an infectious outbreak^53^. The pressure of the pandemic prompted them to utilize coping strategies, which included distraction techniques (exercise, talking to others, engaging in activities) and mental avoidance to keep themselves busy while disengaging from their own plight. Problem-solving strategies such as mastering knowledge of the disease allowed patients to proactively empower themselves and gain control of their health, which minimized their feelings of uncertainty^54^ and improved their mental health^55^. Positive cognitive appraisals of the outbreak situation together with a positive mindset enable one to seizure control of the situation and constructively plan ahead. Some patients re-evaluated the situation based on past outbreaks and experiences in life to anticipate possible scenarios, and assessed the situation to be manageable, which reduced distress^56^. Some patients placed confidence in the government and healthcare systems to manage the outbreak and believed that they would receive good treatment if they needed it^57^. Having faith and trust in the government and health authorities to manage COVID-19 may reduce their fears and perceived vulnerability to the virus^58^. Seeking support from various sources, including family and friends, fellow patients and healthcare providers, are integral in cushioning the psychological complications of the outbreak. The dedicated care and concern shown by healthcare workers positively impacted patients and made them feel supported. All of these factors contribute to enhanced optimism, which favorably alleviates psychological trauma in disasters, and enhances psychological rehabilitation of PTSD^59^.

Guided by findings of this study, which identified the psychological distress and mental health needs of COVID-19 patients, it is paramount for hospitals moving forward, to set up a novel holistic inpatient service with appropriate prevention measures to promote immune and psychological resilience. This is done by catering to the individualized physical and mental health needs of COVID-19 patients who are kept in prolonged isolation. Several principles of this service that could be considered are as follow. (1) Maintain regular physical exercise regimes, especially those that elevate cardiorespiratory fitness at moderate intensity, as they have been found to reduce inflammation and boost immunity^60^. Exercise can be delivered through online instructor-led platforms or via exergaming, which is a hybrid form of physical activity that combines exercise and video games. (2) Develop healthy lifestyle habits with proper nutrition that includes antioxidants, high fiber content, whole grains, and unsaturated fats, which have rapid anti-inflammatory effects that boost immunity, counteract vulnerability to COVID-19 and promote recovery^61^. (3) Educate on proper safety precautions including hand hygiene, wearing of face masks and social distancing to minimize community spread after hospital discharge. (4) Use of online and smartphone-based platforms to deliver various types of psychotherapy to enhance patients’ adaptive and coping capability. An example is trauma-focused-cognitive behavior therapy with emphasis on cognitive reframing of the mindset to help remove unhelpful thoughts about COVID-19 and perceived discrimination, trauma narrative to process personal traumatic experiences during the pandemic, grief therapy to handle potential losses, and relaxation techniques to counteract anxiety, irritability, anger, and PTSD-like symptoms^62^. Sleep hygiene advice can also be provided to improve circadian rhythm and sleep quality. (5) Allow patients the peace of mind to recover from the infection. This includes ensuring data protection and confidentiality of patient details to minimize potential discrimination by others, and ensure affordability of medical care. (6) Provide emotional support to healthcare workers taking care of COVID-19 patients. The mental health of healthcare workers can directly impact the quality of care and psychological well-being of patients. (7) Monitor the association of blood pro-inflammatory cytokine levels with severity of COVID-19 physical and neuropsychiatric symptoms. This may have implications in monitoring disease progression and treatment response, considering the potential bidirectional relationship between COVID-19 and psychiatric disorders. Furthermore, the application of biological markers may better elucidate the yield of using antidepressants as a novel treatment modality for COVID-19.

The present study has merits in being the first study to quantitatively and qualitatively evaluate the neuropsychiatric sequalae and psychological impact of patients with active COVID-19 infection. The qualitative component of the study provides a more personalized account of the perspectives and challenges faced by patients, and strengthens the findings obtained from quantitative measures. The study performed face-to-face interviews as compared with other studies^9^, and was challenging logistically and safety-wise for the interviewers. Nevertheless, this study provided patients with a better interview experience to elicit more authentic information. Furthermore, the comparison of COVID-19 patients to psychiatric patients and healthy individuals in the community provided further clarity that COVID-19 patients had more neuropsychiatric psychiatric symptoms than psychiatric patients and the general population, which could be due to their enhanced pro-inflammatory state and increased stress.

On the other hand, this study has several limitations. First, the sample size of the study was small, and thus results from our study are at risk of Type 2 errors and could not be generalizable to all psychiatric and COVID-19 patients. We were unable to recruit a larger sample owing to the potential risk of infection to the interviewers. Furthermore, the psychiatric and COVID-19 patients whom we recruited had low to moderate severity of their psychiatric condition and stable medical condition without complications, respectively. It would not be ethical and feasible to recruit patients who were psychiatrically and medically unstable. Therefore, our sample participants were not representative of the spectrum of psychiatric disorders and COVID-19 infection. Second, no biological markers such as cytokines were measured to correlate with the neuropsychiatric symptoms. Thus, we were unable to elucidate the relationship between the two, and whether the psychiatric symptoms experienced by COVID-19 patients were the result of the inflammatory effects of COVID-19 infection or owing to other causes such as the psychological effects from being isolated. Third, this study was a cross-sectional study that performed assessments at a particular time point. It would be pertinent to longitudinally review their condition with monitoring of their cytokine levels and neuropsychological data as part of the intervention. Nevertheless, the current data are valuable to provide a preliminary in-depth understanding of the psychological issues faced by COVID-19 patients and serve as a foundation for further studies in this area.

## Conclusion

This study has provided a comprehensive and in-depth exploration of the neuropsychiatric sequalae and psychological impact of acutely infected COVID-19 patients through quantitative and qualitative approaches. COVID-19 patients had higher levels of neuropsychiatric symptoms than psychiatric patients and healthy individuals in the community in terms of impulsivity and insomnia, which could be secondary to their enhanced pro-inflammatory state. COVID-19 patients also experienced emotions including shock, fear, boredom, and hope during their course of treatment, and common concerns about discrimination, medical expenses, care by healthcare workers, and means of self-help behavior were highlighted. The future direction for COVID-19 management involves the setting up of a dedicated holistic service that incorporates preventive measures on exercise, nutrition, safe hygiene practices, psychotherapy, and use of cytokine markers to monitor disease progression and treatment response, coupled with financial and healthcare support.
